# When seconds matter – introducing a surgical salvage strategy by emergency robotic surgery: a review

**DOI:** 10.1186/s13037-026-00483-1

**Published:** 2026-03-21

**Authors:** Ibrahim Büdeyri, Jan Philipp Ramspott, Mazen A. Juratli, Andreas Pascher, Jens Peter Hoelzen

**Affiliations:** Department of General, Visceral and Transplant Surgery, University Hospital Muenster, Muenster, Germany

**Keywords:** Robotic surgery, Intraoperative emergencies, Emergency simulation, Simulation training, Team communication

## Abstract

Robotic surgery has expanded minimally invasive options but introduces specific vulnerabilities during intraoperative emergencies. The physical separation of the surgeon from the patient, restricted access due to docking, and dependence on complex technology can delay life‑saving interventions if teams are not prepared. This review summarizes current evidence and expert practice on emergency management along the perioperative pathway in robotic surgery, focusing on a structured, systems‑based salvage strategy. Key domains include preoperative planning, classification of recoverable and non‑recoverable errors, and standardized protocols for rapid undocking, conversion, hemorrhage control, and resuscitation. Particular emphasis is placed on human factors, including visible name tags, closed‑loop communication, and structured briefings to strengthen situational awareness and role clarity in crises. Simulation‑based curricula and high‑fidelity in situ drills are highlighted as essential for rehearsing rare but high‑impact events such as catastrophic bleeding, cardiorespiratory collapse, or robotic system failure. In parallel, technology‑driven tools such as surgeon‑controlled suction, advanced vessel sealing, and artificial‑intelligence–assisted monitoring are discussed as adjuncts for earlier recognition and standardized documentation of critical events. Integrating these elements into protocolized workflows can improve preparedness, shorten response times, and support safer decision‑making when seconds matter in robotic surgery.

## Introduction

Protecting patient safety in the operating room (OR) hinges on seamless communication and a deeply ingrained safety culture, particularly during the intense pressures of robotic surgery emergencies [1–3]. At its core, communication functions as a two-way exchange where one party conveys information that the recipient grasps without confusion or distortion, though this process proves highly prone to breakdowns that heighten dangers in robot-assisted cases, including device failures or abrupt bleeding episodes.

A landmark study published in 2009 demonstrated the effectiveness of a 19-item World Health Organisation (WHO) - Surgical Safety Checklist (SSC) in diverse hospitals worldwide [4]. Implemented across eight sites with varying resources, the checklist targeted critical phases—pre-anesthesia induction, before incision, and post-procedure—to enhance team communication, consistency, and error prevention in noncardiac surgeries for patients aged 16 and older. Prospective data from over 3,700 patients pre-implementation showed a 1.5% death rate and 11.0% complication rate, which dropped significantly to 0.8% and 7.0%, respectively, after checklist adoption. These reductions, averaging 36% for complications and similar for mortality, underscore the checklist’s role in mitigating preventable surgical harm globally, providing a foundational framework for safety protocols adaptable to advanced contexts like robotic surgery emergencies.

Robotic surgery represents a significant advancement in the field of medical science, offering unprecedented precision, minimized trauma, and reduced recovery times [5]. The integration of robotic systems into surgical practice has enabled surgeons to perform complex procedures with enhanced dexterity and stability [6]. However, as with any medical procedure, emergencies can arise unexpectedly. Given the reliance on sophisticated technology, ensuring effective emergency management in robotic surgery is crucial to guarantee patient safety and optimal surgical outcomes.

Understanding the structure and functionality of robotic surgery is essential for developing effective emergency management strategies [7, 8]. Robotic surgery involves the use of highly advanced systems that typically consist of a surgeon’s console, a patient-side cart equipped with robotic arms, and a high-definition three-dimensional vision system. The surgeon operates the robotic arms, which translate human movements into highly precise actions, reducing the margin of error and allowing for minimally invasive techniques. Despite these advancements, the complexity of robotic systems necessitates robust emergency surgical management protocols.

As robotic surgery continues to evolve, the development of structured emergency response systems becomes essential to ensuring patient safety and preventing avoidable complications. Effective management protocols must integrate advanced simulation training, real-time problem-solving strategies, and coordinated communication to optimize surgical outcomes [9].

This review aims to provide a comprehensive overview of emergency management strategies in robotic surgery, highlighting key risk mitigation techniques, response frameworks, and technological innovations that contribute to a safer and more efficient surgical environment.

## Proactive emergency management in robotic surgery: risk minimization strategies

Robotic surgery enhances precision and ergonomics; however, it also introduces distinct challenges in emergency scenarios. A structured, proactive approach is essential, encompassing strategic planning, risk assessment, and real-time intraoperative salvage strategy.

### Emergency management protocols

To ensure patient safety and minimize procedural disruptions, hospitals and surgical teams must establish comprehensive emergency management protocols. These protocols should be designed to address various potential emergencies and enable rapid, efficient responses. A multi-tiered approach, incorporating technological integration, communication strategies, and team-based interventions, ensures that responses are well-coordinated and effective in high-risk situations. Protocol standardization across institutions further facilitates best-practice implementation and consistency in emergency scenarios.

Bludevich et al. investigate how structured education and checklists affect operating‑room team performance during emergent conversion from robotic to open thoracic surgery [10]. The intervention includes standardized algorithms for conversion, team briefings, stepwise checklists and focused training sessions covering communication of the emergency, stopping the robot, repositioning and performing thoracotomy. After implementation, teams complete critical conversion steps more consistently, with fewer missed actions and better timing, while assessments show improved leadership, communication and situational awareness. The authors conclude that rare conversion events can be managed more safely when supported by rehearsed protocols, simulation and emphasis on non‑technical skills.

Shah et al. present a practical, institutional rapid undocking protocol for da Vinci robotic systems during emergencies, emphasizing time-critical patient access in scenarios like airway compromise, cardiac arrest, uncontrolled hemorrhage, or robotic malfunction [11]. The protocol features a clear trigger phrase (“Emergency undock”) uttered only by the console surgeon (surgical emergencies) or primary anesthesiologist (airway/cardiac issues), initiating a choreographed cascade with predefined roles for all team members including assistant surgeons, nurses, technicians, and anesthesiologists. In-situ simulation drills using the actual robotic cart and mannequin are conducted with formative assessment, didactic review, and summative evaluation, achieving emergency undocking times reduced from 3 min (index case) to 40 s through repeated practice and closed-loop communication. This work demonstrates that standardized protocols with role clarity and regular multidisciplinary rehearsals overcome the unique access limitations of robotic surgery, minimizing delays in life-saving interventions and enhancing team confidence across rare crises. Such findings reinforce the value of protocolized mock-drills in high-volume centers, directly supporting structured preoperative planning, simulation-based training, and interdisciplinary cooperation to optimize patient safety and intraoperative efficiency in robotic procedures.

### Preoperative planning

Thorough preoperative planning is essential to reducing emergency occurrences during robotic surgery. This process involves conducting detailed patient assessments to identify potential risk factors, such as pre-existing medical conditions that could contribute to complications. Additionally, all robotic equipment must undergo rigorous preoperative testing to ensure optimal functionality before surgery begins. Developing a contingency plan for various emergencies, including system malfunctions and power failures, is a crucial component of preoperative preparation. Furthermore, interdisciplinary briefings before each procedure help ensure all team members are familiar with the specific robotic setup and potential risk scenarios. Emergency kits with essential backup instruments should be pre-positioned to allow for rapid deployment when needed.

### Training and simulation

Regular training and simulation exercises are indispensable for preparing surgical teams to handle emergencies in robotic surgery. Team members must be routinely trained in emergency protocols, including manual conversion techniques for cases where robotic system failures necessitate a transition to conventional surgery. Simulation-based training provides an opportunity to practice responses to different emergency scenarios, ensuring that all team members are well-versed in their roles and responsibilities during critical situations. Advanced training modules incorporating virtual reality (VR) and augmented reality (AR) offer enhanced procedural familiarity and facilitate immediate feedback, improving response time and team synchronization. Moreover, recurrent workshops in high-fidelity simulation settings enable real-world adaptation to high-pressure scenarios, reducing cognitive overload during an actual emergency.

Ballas et al. describe a dedicated simulation-based training platform for emergency undocking of the robotic system to rapidly re‑establish full access to the patient in critical situations [12]. High‑fidelity scenarios (for example sudden hemodynamic instability or major bleeding) are used to train teams in the sequence of undocking steps, role allocation and closed‑loop communication. This study demonstrates that structured simulation significantly reduces undocking times and decreases delays and errors during emergencies. Participants also show better role clarity, situational awareness and confidence when managing rare but high‑risk events, underlining emergency undocking training as a key component of robotic safety programs.

Baste et al. report on the introduction of simulation-based emergency training for interprofessional teams involved in robotic and VATS thoracic surgery [13]. Using realistic scenarios such as uncontrolled bleeding, cardiovascular collapse and technical failures, the program trains surgeons, anesthetists and nurses together in both technical management and team behaviors. The training leads to measurable improvements in teamwork, communication, decision‑making and situational awareness, evaluated with validated non‑technical skills rating tools. Participants also report increased confidence in handling rare intraoperative crises, supporting the view that emergency simulation should be an integral, recurring component of robotic and minimally invasive thoracic surgery programs.

Dunnahoo et al. conducted a quality improvement project to develop and implement a standardized emergency robotic undocking protocol (ERUP) through multidisciplinary, in-situ simulation training at a community hospital [14]. Baseline simulations revealed initial delays in undocking times due to unfamiliarity with robotic constraints and team roles during crises like hemorrhage or system failure. The intervention introduced a stepwise ERUP with clear role assignments for surgeons, anesthesiologists, nurses, and technicians, combined with repeated high-fidelity drills in the actual operating room, followed by debriefings to refine communication and critical actions. Post-implementation simulations demonstrated substantial improvements in undocking speed and completion of essential steps, with no changes in self-reported knowledge or confidence but high participant satisfaction with the protocol’s feasibility. These findings highlight the practicality of tailored ERUPs in resource-limited settings, emphasizing simulation’s role in mitigating robotic surgery’s access limitations and supporting your review’s focus on proactive protocol development, team training, and measurable emergency response optimization.

## Challenges in emergency management

### Classification and management of errors in robotic surgery

Surgical emergencies can be categorized into recoverable and non-recoverable errors. Recoverable errors are those that can be rectified intraoperatively without compromising patient safety, such as minor bleeding or instrument malfunctions. In contrast, non‑recoverable errors require immediate intervention to prevent adverse outcomes, including major vascular injuries, cardiac arrest, or unrecoverable failures of the robotic system that mandate conversion or procedure termination. Proper classification of errors facilitates more effective response strategies, allowing teams to prioritize interventions based on urgency and feasibility. Cognitive aids, such as intraoperative decision trees, assist surgeons in systematically diagnosing and addressing errors, enhancing overall emergency resolution efficiency [15].

### Hemorrhage control strategies

Preoperative risk assessment is essential to identify high-risk cases and implement preventive measures. Standardized bleeding control protocols, including vessel clamping, suturing techniques, and the use of hemostatic agents, are necessary components of a comprehensive hemorrhage management strategy [16]. In addition, structured emergency checklists should be readily accessible to ensure that surgical teams can respond promptly to intraoperative bleeding events. Regular competency assessments in advanced hemorrhage management techniques, such as endoscopic vessel sealing and robotic suturing proficiency, ensure that team members are adequately trained to manage severe bleeding scenarios. The integration of artificial intelligence (AI)-assisted hemorrhage detection further enhances early recognition and intervention, reducing the risk of catastrophic blood loss.

### Resuscitation preparedness in robotic surgery

Resuscitation preparedness in robotic surgery requires multiple strategies. Early warning systems, led by anesthesia, facilitate predictive monitoring for hemodynamic instability. Emergency undocking protocols must be structured to enable a rapid and efficient response to critical situations [17]. Furthermore, regular simulation-based resuscitation drills help optimize team coordination and response efficiency. The implementation of standardized emergency algorithms, such as ACLS (Advanced Cardiac Life Support) and REBOA (Resuscitative Endovascular Balloon Occlusion of the Aorta), enables teams to respond rapidly to circulatory collapse. Additionally, communication optimization through closed-loop techniques ensures that resuscitation commands are clearly received and executed without ambiguity [18].

Huser et al. developed a high-fidelity, in-situ simulation to assess multidisciplinary team performance during a life-threatening emergency in robot-assisted surgery [19]. Complete operating room teams managed a simulated cardiorespiratory collapse during a mock robotic procedure, requiring rapid recognition, communication, undocking or repositioning, and initiation of advanced resuscitation measures. After structured training and repetition, teams achieved resuscitation within seconds despite robotic constraints, with significantly faster completion of critical steps like chest compressions and defibrillation, plus improved adherence to algorithms. This study underscores that rare but catastrophic events in robotic surgery demand specific preparation due to restricted patient access, and demonstrates that repeated, realistic simulations markedly enhance emergency response times, coordination, and team confidence. Such findings support integrating regular multidisciplinary emergency drills into robotic training programs to optimize intraoperative decision-making and patient safety.

### Robot‑related mechanical and system failures

Mechanical failure of robotic instruments, including jaw fracture or detachment of small end‑effector components, constitutes an important subgroup of device‑related complications in robotic surgery [20, 21]. Reported events include stuck or broken needle drivers and separation of scissors tips or jaw covers, in some cases with fragments remaining unrecognized in the operative field until postoperative imaging or case review [20, 21]. Such malfunctions can interrupt critical steps, compromise precise tissue handling, and when fragments are not retrieved, result in unretrieved device material that may prompt conversion, additional retrieval procedures, or postoperative surveillance [21]. In addition to instrument‑level problems, non‑recoverable failures of the robotic system itself, such as persistent system errors, loss of console–cart communication, or arm malfunction that cannot be resolved intraoperatively, may require conversion to an alternative approach or termination of the procedure [21, 22]. Mitigation strategies include routine visual inspection of instrument jaws at each exchange and at the end of the case, documentation of all detachable components, and predefined algorithms for troubleshooting, fragment localization and retrieval, and timely conversion when a system error persists [20, 22]. These measures can be reinforced through team training and simulation scenarios that specifically address both instrument‑failure and system‑failure events under time pressure [21, 22].

## Interdisciplinary cooperation: team integration and innovative strength in robotic surgery

### Enhancing team communication: the role of name tags in OR efficiency

Clear and effective communication among surgeons, anesthesiologists, surgical nurses, and anesthesia nurses remains critical for ensuring high-quality, safe, and efficient perioperative care, particularly in the complex environment of robotic surgery emergencies where technical and human factors intersect. Despite widespread adoption of the WHO-SSC, studies by van Dalen et al. [23] and Birnbach et al. [24] reveal persistent gaps, with the majority of OR team members still unaware of each other’s names. This issue of poor name memorability, recognized for decades, underscores ongoing challenges in fostering true team familiarity [25].

Inadequate team communication continues to rank as a primary contributor to patient safety lapses in the OR [26, 27], accounting for up to 56% of adverse intraoperative and postoperative events in surgical settings, often resulting in severe outcomes like death or permanent functional loss [26]. In robotic surgery emergencies, these communication failures can amplify risks due to the steep learning curve, equipment dependencies, and urgency of scenarios like intraoperative bleeding or robot failure, emphasizing the need for enhanced SSC adaptations such as role-specific drills and real-time name verification to mirror the checklist’s proven global impact on error reduction [28, 29].

Effective communication is integral to emergency response in robotic surgery. Standardized name tags enhance role clarity, minimize miscommunication, and improve overall patient safety. By ensuring clear identification of team members, name tags facilitate streamlined intraoperative coordination, particularly in high-stress situations where precise communication is essential. Communication training programs that emphasize structured handoffs and real-time situational awareness contribute to improved team dynamics and reduce the risk of misunderstandings that could delay emergency interventions.

Bungert et al. conducted a proof-of-concept study introducing individual name tags in the operating room to enhance interprofessional communication among constantly changing teams, with particular relevance to robotic surgery where spatial separation, noise, and microphone-based interactions complicate direct exchanges [30]. Staff across surgical, anesthesia and nursing roles wore tags displaying names and professions during procedures; post-implementation surveys (median rating 3.4/5) revealed strong positive feedback, especially for general communication, direct colleague contact, and task delegation, with over 90% adherence and surgeons/nurses reporting the greatest benefits. The intervention reduced communication barriers, hierarchies, and hesitations toward unfamiliar colleagues, fostering closed-loop communication (e.g., confirming receipt of instructions) and potentially improving patient safety by minimizing errors from misunderstandings. These findings directly support the role of simple, low-cost tools like name tags in robotic settings to promote team familiarity, role clarity, and efficient emergency coordination, warranting broader adoption and further longitudinal studies on clinical outcomes.

### Technology-driven solutions for emergency management

Technological advancements, such as surgeon-controlled suction, automated table motion, and enhanced visualization systems, contribute to greater surgical autonomy. The integration of AI-driven monitoring systems can further enhance real-time decision-making, allowing for predictive risk assessments and early intervention strategies. Continued collaboration with medical technology developers is essential to refining these systems and improving patient outcomes. The incorporation of real-time performance analytics and machine learning-driven procedural optimization offers new avenues for reducing intraoperative risks and standardizing emergency responses across institutions.

AI in robotic surgery refers to machine‑learning methods, often based on deep‑learning, that analyze perioperative data such as endoscopic video, robotic instrument kinematics and physiological parameters [31, 32]. In current systems, AI is mainly implemented as computer‑vision tools that perform automated structure and instrument recognition, instrument tracking, and procedural phase detection, providing additional quantitative information alongside the surgeon’s own assessment [31–33]. These models can be used to monitor specific steps of an operation, detect deviations from expected patterns (for example, onset or progression of bleeding), and document timing and sequence of key actions during emergency management [31, 32]. In the context of emergencies, such applications are best regarded as decision‑support. They can assist with earlier recognition and more standardized documentation of critical events, but the surgeon and anesthesiology team remain responsible for interpretation and final decisions [31–33].

## Conclusion

A structured, proactive approach to emergency management remains central to safe robotic surgery. Aligning clear conceptual frameworks with high fidelity simulation, standardized emergency protocols, and technology supported monitoring can improve preparedness, shorten response times, and limit preventable harm during intraoperative emergencies. Strengthening communication practices, particularly explicit role allocation and consistent closed loop exchanges, further supports coordinated action when seconds matter. Future development of robotic programs should embed emergency surgical management into core program design, with routine multidisciplinary drills, systematic evaluation of undocking and conversion workflows, and carefully implemented AI-based decision support tools. Sustained collaboration across surgical, anesthesia, nursing, and engineering teams can foster resilient systems that maintain patient safety during rare but high impact emergencies and enhance the overall reliability of minimally invasive care.

## Data Availability

No datasets were generated or analysed during the current study.

## Abbreviations

OR: Operating room
WHO: World Health Organization
SSC: Surgical Safety Checklist
VR: Virtual reality
AR: Augmented reality
ERUP: Emergency robotic undocking protocol
VATS: Video-assisted thoracoscopic surgery
AI: Artificial intelligence
ACLS: Advanced Cardiac Life Support
REBOA: Resuscitative Endovascular Balloon Occlusion of the Aorta

## References

[1] Nagpal K, Vats A, Lamb B, Ashrafian H, Sevdalis N, Vincent C, et al. Information transfer and communication in surgery: a systematic review. Ann Surg. 2010;252(2):225–39. 20647929 10.1097/SLA.0b013e3181e495c2

[2] Neuhaus C, Huck M, Hofmann G, St. Pierre M, Weigand MA, Lichtenstern C. Applying the human factors analysis and classification system to critical incident reports in anaesthesiology. Acta Anaesthesiol Scand. 2018;62(10):1403–11. 29974938 10.1111/aas.13213

[3] Weldon S-M, Korkiakangas T, Bezemer J, Kneebone R. Communication in the operating theatre. J Br Surg. 2013;100(13):1677–88. 10.1002/bjs.9332 24227352

[4] Haynes AB, Weiser TG, Berry WR, Lipsitz SR, Breizat A-HS, Dellinger EP, et al. A surgical safety checklist to reduce morbidity and mortality in a global population. N Engl J Med. 2009;360(5):491–9. 19144931 10.1056/NEJMsa0810119

[5] Reddy K, Gharde P, Tayade H, Patil M, Reddy LS, Surya D. Advancements in Robotic Surgery: A Comprehensive Overview of Current Utilizations and Upcoming Frontiers. Cureus. 2023;15(12):e50415. 38222213 10.7759/cureus.50415 PMC10784205

[6] Riad A, Hadid M, Elomri A, Al-Ansari A, Rejeb MA, Qaraqe M, et al. Advancements and challenges in robotic surgery: A holistic examination of operational dynamics and future directions. Surg Pract Sci. 2025;22:100294. 40697312 10.1016/j.sipas.2025.100294 PMC12280407

[7] Lunardi N, Abou-Zamzam A, Florecki KL, Chidambaram S, Shih I-F, Kent AJ, et al. Robotic technology in emergency general surgery cases in the era of minimally invasive surgery. JAMA Surg. 2024;159(5):493–9. 38446451 10.1001/jamasurg.2024.0016 PMC10918578

[8] Milone M, Anoldo P, De’Angelis N, Coccolini F, Khan J, Kluger Y, et al. The role of RObotic surgery in EMergency setting (ROEM): protocol for a multicentre, observational, prospective international study on the use of robotic platform in emergency surgery. World J Emerg Surg. 2024;19(1):20. 38835071 10.1186/s13017-024-00542-x PMC11149189

[9] Kalipershad SN, Peristerakis I. The introduction of an emergency safety protocol coupled with simulation training in robotic surgery, has enabled a more cohesive and efficient response to emergencies. Surgeon. 2022;20(3):151–6. 33947630 10.1016/j.surge.2021.03.007

[10] Bludevich B, Dickson KM, Reddington H, Lim CJ, Hazeltine M, Buettner H, et al. Emergent robotic surgery conversions: improving operating room team performance through high fidelity simulations. J Thorac Disease. 2024;16(7):4286. 39144341 10.21037/jtd-24-291 PMC11320252

[11] Shah SB, Chawla R, Rawal SK. Rapid undocking protocol for the da Vinci surgical robot during emergency situations. Indian J Anaesth. 2023;67(4):398–400. 37303879 10.4103/ija.ija_946_22 PMC10248886

[12] Ballas D, Cesta M, Roulette GD, Rusnak M, Ahmed R. Emergency undocking in robotic surgery: a simulation curriculum. J Vis Exp. 2018(135):57286. 10.3791/57286 PMC6101302 29863667

[13] Baste J-M, Bottet B, Selim J, Sarsam M, Lefevre-Scelles A, Dusseaux M-M, et al. Implementation of simulation-based crisis training in robotic thoracic surgery: how to improve safety and performance? J Thorac Disease. 2021;13(Suppl 1):S26. 34447589 10.21037/jtd-2020-epts-03 PMC8371544

[14] Dunnahoo FC, Thompson J, Muckler VCS. Developing an emergency robotic undocking protocol using simulation. J Interprofessional Educ Pract. 2021;24:100464.

[15] Greig PR, Zolger D, Onwochei DN, Thurley N, Higham H, Desai N. Cognitive aids in the management of clinical emergencies: a systematic review. Anaesthesia. 2023;78(3):343–55. 36517981 10.1111/anae.15939 PMC10107924

[16] AlMasri S, Kraftician J, Zureikat A, Paniccia A. Management of Intra-Operative Hemorrhage and Safe Venous Resection in Robotic-Assisted Pancreaticoduodenectomy: Techniques to Avoid Open Conversion. J Gastrointest Surg. 2023;27(8):1753–6. 37101091 10.1007/s11605-023-05684-y PMC11225575

[17] Melnyk R, Saba P, Holler T, Cameron K, Mithal P, Rappold P, et al. Design and Implementation of an Emergency Undocking Curriculum for Robotic Surgery. Simul Healthc. 2022;17(2):78–87. 34387245 10.1097/SIH.0000000000000596

[18] Lee H, Merry AF, Woodward-Krohn R, Weller JM. Use and effectiveness of directed, closed-loop communication in the operating theatre: mixed methods analysis of simulated clinical emergencies. Br J Anaesth. 2025;135(5):1279–85. 40537326 10.1016/j.bja.2025.05.015 PMC12597367

[19] Huser A-S, Müller D, Brunkhorst V, Kannisto P, Musch M, Kröpfl D, et al. Simulated life-threatening emergency during robot-assisted surgery. J Endourol. 2014;28(6):717–21. 24471449 10.1089/end.2013.0762

[20] Singh S, Bora GS, Devana SS, Mavuduru RS, Singh SK, Mandal AK. Instrument malfunction during robotic surgery: A case report. Indian J Urol. 2016;32(2):159–60. 27127362 10.4103/0970-1591.174781 PMC4831508

[21] Alemzadeh H, Raman J, Leveson N, Kalbarczyk Z, Iyer RK. Adverse Events in Robotic Surgery: A Retrospective Study of 14 Years of FDA Data. PLoS ONE. 2016;11(4):e0151470. 27097160 10.1371/journal.pone.0151470 PMC4838256

[22] Agcaoglu O, Aliyev S, Taskin HE, Chalikonda S, Walsh M, Costedio MM, et al. Malfunction and failure of robotic systems during general surgical procedures. Surg Endosc. 2012;26(12):3580–3. 22678175 10.1007/s00464-012-2370-9

[23] van Dalen ASH, Swinkels JA, Coolen S, Hackett R, Schijven MP. Improving teamwork and communication in the operating room by introducing the theatre cap challenge. J Perioper Pract. 2022;32(1–2):4–9. 35001734 10.1177/17504589211046723 PMC8750134

[24] Birnbach DJ, Rosen LF, Fitzpatrick M, Paige JT, Arheart KL. Introductions during time-outs: Do surgical team members know one another’s names? Joint Comm J Qual Patient Saf. 2017;43(6):284–8. 10.1016/j.jcjq.2017.03.001 28528622

[25] Cohen G, Faulkner D. Memory for proper names: Age differences in retrieval. Br J Dev Psychol. 1986;4(2):187–97.

[26] Goldhaber NH, Reeves JJ, Puri D, Berumen JA, Tran M, Clay BJ, et al. Surgery and anesthesia preoperative virtual huddle: a pilot trial to enhance communication across the drape. Appl Clin Inf. 2023;14(04):772–8. 10.1055/s-0043-1772687 PMC10533219 37758227

[27] Yates M, Foran P. Improving perioperative communication: Can labelled theatre caps play a role? J Perioperative Nurs. 2023;35(5):e–37.

[28] Etherington C, Wu M, Cheng-Boivin O, Larrigan S, Boet S. Interprofessional communication in the operating room: a narrative review to advance research and practice. Can J Anesthesia/Journal canadien d’anesthésie. 2019;66(10):1251–60. 10.1007/s12630-019-01413-9 31140044

[29] Manser T. Teamwork and patient safety in dynamic domains of healthcare: a review of the literature. Acta Anaesthesiol Scand. 2009;53(2):143–51. 19032571 10.1111/j.1399-6576.2008.01717.x

[30] Bungert AD, Ramspott JP, Szardenings C, Knipping A, Struecker B, Pascher A, et al. The Power of The (First) Name: Do name tags for operating room staff improve effective communication and patient safety? A proof-of-concept study from an academic medical center in Germany. Patient Saf Surg. 2024;18(1):35. 39654060 10.1186/s13037-024-00418-8 PMC11629488

[31] Vasey B, Lippert KAN, Khan DZ, Ibrahim M, Koh CH, Layard Horsfall H, et al. Intraoperative Applications of Artificial Intelligence in Robotic Surgery: A Scoping Review of Current Development Stages and Levels of Autonomy. Ann Surg. 2023;278(6):896–903. 36177855 10.1097/SLA.0000000000005700 PMC10631501

[32] Knudsen JE, Ghaffar U, Ma R, Hung AJ. Clinical applications of artificial intelligence in robotic surgery. J Robot Surg. 2024;18(1):102. 38427094 10.1007/s11701-024-01867-0 PMC10907451

[33] Iftikhar M, Saqib M, Zareen M, Mumtaz H. Artificial intelligence: revolutionizing robotic surgery: review. Ann Med Surg (Lond). 2024;86(9):5401–9. 39238994 10.1097/MS9.0000000000002426 PMC11374272

